# Local Duplication of Gonadotropin-Releasing Hormone (GnRH) Receptor before Two Rounds of Whole Genome Duplication and Origin of the Mammalian GnRH Receptor

**DOI:** 10.1371/journal.pone.0087901

**Published:** 2014-02-03

**Authors:** Fatemeh Ameri Sefideh, Mi Jin Moon, Seongsik Yun, Sung In Hong, Jong-Ik Hwang, Jae Young Seong

**Affiliations:** 1 Graduate School of Medicine, Korea University, Seoul, Republic of Korea; 2 Department of East-West Integrated Medicine, College of Oriental Medicine, Gachon University, Incheon, Republic of Korea; University of Rouen, France, France

## Abstract

Gonadotropin-releasing hormone (GnRH) and the GnRH receptor (GnRHR) play an important role in vertebrate reproduction. Although many GnRHR genes have been identified in a large variety of vertebrate species, the evolutionary history of GnRHR in vertebrates is unclear. To trace the evolutionary origin of GnRHR we examined the conserved synteny of chromosomes harboring GnRHR genes and matched the genes to linkage groups of reconstructed vertebrate ancestor chromosomes. Consistent with the phylogenetic tree, three pairs of GnRHR subtypes were identified in three paralogous linkage groups, indicating that an ancestral pair emerged through local duplication before two rounds of whole genome duplication (2R). The 2R then led to the generation of six subtypes of GnRHR. Some subtypes were lost during vertebrate evolution after the divergence of teleosts and tetrapods. One subtype includes mammalian GnRHR and a coelacanth GnRHR that showed the greatest response to GnRH1 among the three types of GnRH. This study provides new insight into the evolutionary relationship of vertebrate GnRHRs.

## Introduction

Families of neuropeptides and receptors have emerged through evolutionary processes such as gene/chromosome duplications. In particular, two rounds (2R) of whole genome duplication (WGD) during early vertebrate evolution contributed to the expansion of family members [1]–[8]. Moreover, teleost fish underwent a teleost-specific third round (3R) of genome duplication [6]. In addition to WGD, local tandem duplication of some genes and loss of redundant genes occurred before and after 2R [9]–[14], therefore the evolutionary scheme of a gene family can be highly complicated. Genome duplication events have produced paralogous chromosomal regions, also called paralogons, that provide a basis for exploring orthologous and paralogous relationships among gene family members as well as predicting gene loss in the paralogons [1], [2], [3], [14], [15]. In addition, tracing the family of genes on reconstructed pre-2R vertebrate ancestral chromosomes (*VAC*) is a fast and relatively accurate way to explore relationships among members of a family that contains a large number of paralogous genes [14], [16]. In particular, because this method provides a WGD scheme for each ancestral linkage group, gene duplication and/or loss during each step of genome duplication can be traced [16].

The neuropeptide gonadotropin-releasing hormone (GnRH) and its G protein-coupled receptor GnRHR play a pivotal role in the control of reproduction in vertebrates [17], [18]. To date, many GnRH and GnRHR genes have been identified in invertebrate and vertebrate species using conventional biochemical and bioinformatics tools [13], [19]–[28]. Most vertebrate species have two or three forms of GnRH in the brain (GnRH1, GnRH2, and GnRH3) [13], [19], [20], [29]. The corresponding genes are likely to have emerged through 2R because the three subtypes of the GnRH gene are on three different paralogons that share common neighbor gene families [13]. Most vertebrate species also possess multiple forms of GnRHR. For example, teleost fish possess four or five isoforms of GnRHR [25], [29], whereas amphibian and reptilian species have three forms of GnRHR [23], [30]. In our previous study using synteny analysis, we proposed four vertebrate GnRHR clades: non-mammalian GnRHR n1, n2, and n3, and mammalian GnRHR m1. GnRHR n1 and n2 were suggested to have emerged through local duplication before 2R because these genes are on the same linkage group of vertebrate chromosomes [13].

In an extension of our previous study, the present study examined more diverse vertebrate and invertebrate species including lamprey (*Petromyzon marinus*), coelacanth (*Latimeria chalumnae*), spotted gar (*Lepisosteus oculatus*), *Ciona intestinalis*, amphioxus (*Brnachiostoma floridae*), *Caenorhabditis elegans*, and *Drosophila melanogaster*. Synteny analysis and relocation of GnRHR genes and their neighboring genes on vertebrate ancestral linkage groups revealed that three pairs of GnRHRs reside on three paralogous chromosomal regions. When combined with phylogenetic analysis, this observation suggests the presence of six subtypes of vertebrate GnRHRs that arose through local duplication on the same chromosome followed by 2R. The fourth pair of GnRHR was probably lost during 2R. In addition, our study suggests that a coelacanth GnRHR is an ortholog of mammalian GnRHR because the proteins share a high degree of amino acid sequence identity and both receptors show the highest activity in response to GnRH1 among three GnRH types, thus resolving an enduring question regarding the origin of mammalian GnRHR.

## Results

### Phylogenetic analysis of GnRHR and evolutionary history of the vertebrate *GnRHR* genes

**Figure 1:**
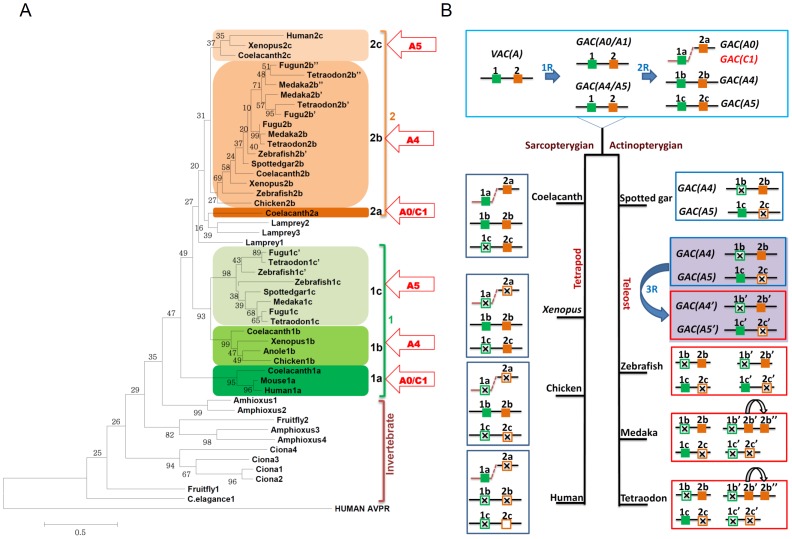
Phylogenetic tree construction for GnRHR-like receptors and evolutionary relationship of vertebrate GnRHRs genes. ***A***, Amino acid sequences of vertebrate species (human, mouse, chicken, anole lizard, *Xenopus*, coelacanth, spotted gar, zebrafish, medaka, fugu, stickleback, tetraodon, and lamprey) and invertebrate species (*C. elegans*, *Drosophila*, *Ciona*, and amphioxus) were aligned using MUSCLE and the phylogeny tree was generated by MEGA 5.05. The human arginine vasopressin receptor 1a protein sequence was used as an outgroup. The vertebrate GnRHRs are grouped monophylogetically. The vertebrate gene types 1 and 2 are illustrated by green and orange, respectively. The arrows indicate the location of each vertebrate gene type on the ancestral linkage group. ***B***, Prior to 2R, GnRHR types 1 and 2 emerged as a product of local duplication on the *VAC A*. Following 2R, a, b, and c subtypes of each ancestral type 1 and 2 were produced on *GAC A0, GAC A4*, and, *GAC A5* respectively and GnRHR type 1a was translocated to *GAC C1*. During the evolution of vertebrate species some GnRHR subtypes have been lost or/and duplicated. The empty box indicates a pseudogene in a human chromosome. An empty box with a cross indicates loss of the gene.

The amino acid sequences of GnRHR from various vertebrate taxa including 13 species (human, mouse, anole lizard, chicken, *Xenopus*, coelacanth, spotted gar, zebrafish, medaka, fugu, stickleback, tetraodon, and lamprey) (Table 1) and invertebrate taxa including 4 species (*C. elegans*, *Drosophila*, *Ciona*, and amphioxus) (Table S1) were retrieved from ENSENBL and NCBI databases. Phylogenetic analysis reveals that vertebrate GnRHRs are grouped monophylogenetically excluding invertebrate GnRHR-like receptors (Figure 1A). To explore the evolutionary relationship among the vertebrate GnRHR lineage, we examined the location of each GnRHR lineage on reconstructed linkage groups of vertebrate ancestors proposed by Nakatani et al. [6]. The Nakatani model suggests the presence of 10–13 pre-2R vertebrate ancestral chromosomes (*VAC*), defined as the *A-J* linkage groups. These *VAC*s then underwent 2R to generate approximately 40 post-2R gnathostome ancestor chromosomes (*GACs A0-J1*). Because chromosomal segments of medaka, chicken, mouse, and human have been matched to these reconstructed linkage groups, the location of a current gene can be traced back to the linkage groups [14], [16]. Our data showed that three pairs of GnRHR subtypes (1a–2a, 1b–2b, and 1c–2c) are found in three related *GAC* linkage groups, *GAC A0/C1*, *A4*, and *A5* (Figure 1A). Based on phylogenetic analysis and the gene location on *GACs*, we propose that vertebrate GnRHRs originated from two ancestral *GnRHR* genes designated types 1 and 2 that emerged through a local duplication on the same chromosome before 2R. During 2R each ancestor gene gave rise to subtypes ‘a’ on *GAC A0/C1*, ‘b’ on *GAC A4*, and ‘c’ on *GAC A5*, resulting in six subtypes (1a,b,c and 2a,b,c) of GnRHR in vertebrates. The subtype 1a includes mammalian GnRHR and a coelacanth GnRHR. No ray-finned fish (actinopterygii including spotted gar) GnRHRs belong to the 1a subtype. The subtypes 1b and 1c are included in the same branch. The 1b subtype includes tetrapod and lobe-finned fish (sarcopterygii) receptors whereas the 1c subtype contains only ray-finned fish receptors. This observation may lead to misunderstanding of the origin of these subtypes. For example, 1b and 1c subtype receptors might be considered to be orthologous. However, as 1b and 1c subtype receptors are on different *GACs* (*GAC A4* and *A5*, respectively) they are more likely to be paralogs generated through a second round of WGD. Therefore, it seems likely that the 1b subtype was lost in ray-finned fish and the 1c subtype was lost in lobe-finned fish after 2R. The teleost-specific 3R may have generated an additional form, the 1c′ subtype. The 2a subtype includes only a coelacanth receptor therefore this subtype was lost in other vertebrates. The 2b subtype includes both ray-finned and lobe-finned fish receptors. The evolution of the 2b subtype in teleosts is highly complicated. The teleost-specific 3R might have contributed to generation of the 2b′ subtype, which was followed by local duplication for emergence of the 2b″ subtype. The 2c subtype is composed of human, *Xenopus,* and coelacanth receptors, but no ray-finned fish receptors (Figure 1A).

**Table 1:** Classification of vertebrate GnRHR based on phylogenetic and synteny analysis.

Vertebrate species			Subtype (GAC)			
	1a (A0/C1)	1b (A4)	1c (A5)	2a (A0/C1)	2b (A4)	2c (A5)
**Human** *(H. sapiens)*	**GnRHR (m1)** AAI13547 (Ch4)					**GnRHR (n3/m2)** NR_002328 (Ch1)
**Mouse** *(M. musculus)*	**GnRHR (m1)** NP_034453 (Ch7)					
**Anole lizard** *(A. carolinensis)*		**GnRHR (n2)** ENSACAP00000004352 (Scaffold:GL343392)				
**Chicken** *(G. gallus)*		**GnRHR (n2)** NP_989984 (Ch10)			**GnRHR (n1)** NP_001012627 (Ch10)	
**Western clawed frog** *(X. tropicalis)*		**GnRHR (n2)** NP_001107548 (Scaffold:GL172999)			**GnRHR (n1)** NP_001107547 (Scaffold: GL172658)	**GnRHR (n3/m2)** NP_001107549 (Scaffold:GL176594)
**Coelacanth** *(L.chalumnae)*	**GnRHR1a** ENSLACG00000002185 (Scaffold:JH130998)	**GnRHR1b** ENSLACG00000016584 (Scaffold:JH126619)		**GnRHR2a** ENSLACG00000013490 (Scaffold:JH127011)	**GnRHR2b** ENSLACG00000018198 (Scaffold:JH126611)	**GnRHR2c** ENSLACG00000014236 (Scaffold:JH126664)
**Spotted gar** *(L.oculatus)*			**GnRHR** ENSLOCG00000008760 (Ch LG24)		**GnRHR** ENSLOCG00000014360 (Ch LG3)	
**Zebrafish** *(D. renio)*			**GnRHR (n3)** NP_001170921 (Ch16)		**GnRHR (n2)** NP_001138451.1 (Ch7)	
			**GnRHR′ (n3b)** NP_001138452 (Scaffold:Zv-NA22)		**GnRHR′ (n1)** NP_001091663 (Ch18)	
**Medaka** *(O. latipes)*			**GnRHR (n3)** NP_001098392 (Scaffold:2457)		**GnRHR (n1)** NP_001098393 (Ch6)	
					**GnRHR′ (n2)** ENSORLP00000015859 (Scaffold:1670)	
					**GnRHR″ (n1b)** NP_001098352 (Ch3)	
**Green pufferfish** *(T. nigroviridis)*			**GnRHR (n3)** BAE45702 (Ch_Unrandom)		**GnRHR (n1)** BAE45698 (Ch13)	
			**GnRHR′ (n3b)** BAE45700 (Ch17)		**GnRHR′ (n2)** ENSORLP00000015859 (Scaffold:1670)	
					**GnRHR″ (n1b)** BAE45694 (ChUnrandom)	
**Stickleback** *(G.aculeatus)*			**GnRHR (n3)** ENSGACP00000004101 (Ch20)		**GnRHR (n1)** ENSGACP00000014249 (Ch19)	
			**GnRHR′ (n3b)** ENSGACP00000000651 (Scaffold:101)		**GnRHR′ (n2)** ENSGACP00000021774 (Ch2)	
					**GnRHR″ (n1b)** ENSGACP00000019583 (Ch2)	
**Fugu** *(T. rubripes)*			**GnRHR(n3)** ENSTRUP00000014399 (Scaffold:270)		**GnRHR (n1)** ENSTRUP00000019152 (Scaffold:217)	
			**GnRHR′ (n3b)** ENSTRUP00000005457 (Scaffold:291)		**GnRHR′ (n2)** ENSTRUP00000014430 (Scaffold:174)	
					**GnRHR″ (n1b)** ENSTRUP00000018665 (Scaffold:92)	
**Lamprey** (*P. marinus*)				**GnRHR1** ENSPMAP00000010120 (Scaffold:GL493405)	**GnRHR2** ENSPMAP00000003609 (Scaffold:GL483393)	**GnRHR3** ENSPMAP00000011312 (Scaffold:GL483841)

The subtype annotations were based on phylogeny and location on gnathostome ancestor gene linkage blocks *GAC A0/C1, GAC A4*, and *GAC A5*. The accession numbers for NCBI and ENSEMBL and gene location are given below the names of the receptors. The receptor names according to our previous classification [13] are shown in parentheses. The five new coelacanth GnRHRs follow current subtype annotation. ′, product of 3R; ″, product of a local duplication after 3R.

Based on these results, we propose an evolutionary history of vertebrate GnRHRs. Type 1 and 2 receptors may have emerged through a local duplication on *VAC A* before 2R. During the first round (1R) of WGD, two genes were duplicated on *GAC A0/A1* and *A4/5* linkage groups. According to the Nakatani model, *GAC A2/3* separated from the *GAC A4/5* linkage group by fission after 1R [6] therefore *GAC A2/3* is not discussed here. The *GAC A4/5* linkage group doubled during the second round (2R) of WGD to produce pairs of *GnRHR* on *GACs A4* and *A5*. The second round of WGD may not have generated the fourth GnRHR pair on *GAC A1*. Alternatively, the fourth GnRHR pair on *GAC A1* might have been lost after 2R. After 2R, the 1a subtype gene appears to have moved to the *GAC C1* linkage group, probably through chromosomal rearrangement or a single gene translocation (Figure 1B). Thus, gnathostome ancestors may have six subtypes of GnRHR. Some subtypes of GnRHR have been lost after the divergence of ray-finned and lobe-finned fish. For example, coelacanth lost the 1c subtype and *Xenopus* lacks 1a, 2a, and 1c subtypes. Chicken has only two subtypes, 1b and 2b, and humans possess one functional receptor of the 1a subtype and one nonfunctional pseudogene of the 2c subtype [24]. In early actinopterygians, GnRHRs on *GAC A0/C1* are likely to have disappeared. Thus, spotted gar contains only two GnRHRs (2b and 1c) on *GAC A4* and *A5*. However, GnRHRs on *GACs A4* and *A5* were duplicated through the teleost-specific 3R, followed by loss of some subtypes. Zebrafish contains four subtypes of receptors, 2b, 2b′, 1c, and 1c′. In medaka and tetraodon, the 2c′ subtype was lost but a 2b″ subtype emerged, probably through a local duplication (Figure 1B). Although we propose a scenario for a doublet GnRHR before 2R, a triplet GnRHR before 2R is also possible because the 1a subtype is phylogenetically distinct from 1b and 1c subtypes. Such a scenario involves an additional local duplication, generating three types (1a, 1b/c, and 2a/b/c) before 2R.

### Synteny of vertebrate chromosomes containing GnRHRs

**Figure 2:**
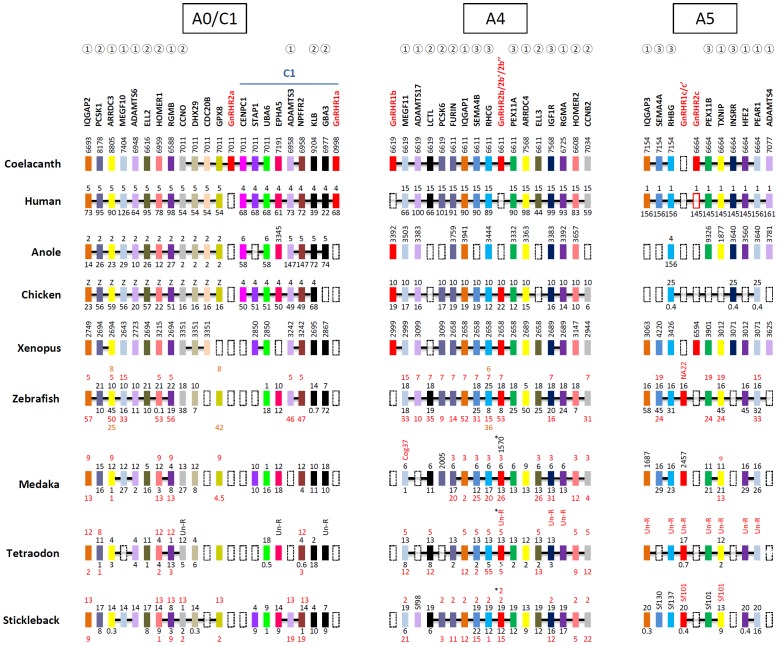
Synteny analysis for chromosome fragments harboring vertebrate GnRHRs. GnRHR and neighboring genes in human, coelacanth, anole, chicken, *Xenopus*, zebrafish, medaka, tetraodon, and stickleback were matched on ancestral linkage groups. The GnRHRs are illustrated in red. Orthologous genes are aligned in the same column and indicated by the same color. The chromosome number and gene location (in megabases) are indicated above and below the indicated genes respectively. For genes located on scaffolds, the gene positions are indicated vertically with only the last four digits of the scaffold number (*e.g*., JH127011 in coelacanth is indicated as 7011). For teleost species, the duplicated genes are represented by two lines of numbers because of the third round of whole genome duplication. The broken boxes indicate loss or absence of genes. The common paralogs across *GAC A0, GAC A4, GAC A5*, and *GAC C1* are indicated by open circles containing numbers as follows: 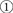 indicates the gene families found on *GACs A0, A4*, and *A5*; 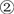 indicates the gene families observed in *GACs A0* and *A4*; 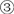 indicates the genes in *GACs A4* and *A5*. In the case of stickleback and medaka, (*) indicates that 2b″ was generated from local gene duplication of 2b′.

To corroborate the gene/genome duplication scheme of the vertebrate GnRHR family, we performed synteny analysis of chromosomal fragments containing GnRHRs. The coelacanth scaffold JH127011 contains the GnRHR 2a subtype and its neighboring genes, which are also found in regions of human chromosomes 5 and 4 that fall into *GAC A0* and *C1*, respectively. It is noteworthy that this region of chromosome 4 contains the GnRHR 1a subtype (Figure 2). Unfortunately, 1a and 2a subtypes are not found in other vertebrate species. The presence of neighbor genes belonging to either *GAC A0* or *C1* in the coelacanth scaffold JH127011 suggests the possibility that the linkage groups including *GAC A0* and *C1* were previously linked. This possibility is partly supported by observations that the paralogous genes *ADAMTS6* and *ADAMTS3* are on either *GAC A0* or *C1* and that some genes in fish, including *ADAMTS3*, *EPHA5*, and *NPFFR2* on *GAC C1*, are on the same chromosomal regions as genes that map on *GAC A0* (Figure 2). An alternative scenario is that GnRHR 1a and some neighbors were translocated to the *GAC C1* linkage group.

The GnRHR 1b and 2b subtypes are on coelacanth scaffolds JH126619 and JH126611, respectively. Neighbor genes on these two scaffolds are found on the same chromosomes in human (chromosome 15) and chicken (chromosome 10). The 1b subtype is absent in teleosts. The teleost-specific 3R might have contributed to doubling of the 2b subtype to produce 2b and 2b′ subtypes as these subtypes are found in chromosomal regions that seem to have been duplicated through 3R [6]. In addition, medaka, tetraodon, and stickleback have an additional subtype 2b″ that is likely to have emerged through a local duplication after 3R. In stickleback, the 2b′ and 2b″ subtypes are on the same chromosome (Figure 2). This observation is consistent with the phylogenetic tree (Figure 1A).

Chromosomal regions with GnRHR 1c or 2c subtypes share neighbors in vertebrate chromosomal regions. The 2c subtype is present in coelacanth, human, and *Xenopus*, but not in ray-finned fish. In contrast, the 1c subtype is present in ray-finned fish but absent in lobe-finned fish and tetrapods. There are two 1c subtype genes in teleost fish. As one gene is on a short scaffold with few neighbors, it is unclear whether these genes were generated by the teleost 3R or local duplication (Figure 2).

Many paralogous genes were shared between these linkage groups (Figure 2). For example, *IQGAP*, *ADAMTS*, *MEGF/PEAR*, *RGM/HFE*, and *ARRDC/TXNIP* family genes are commonly present in the three linkage groups. Paralogous genes for *PCSK1, ELL2, HOMER1*, and *CCNO* on *GAC A0* are present in *GAC A4* and paralogous genes for *SEMA4B, RHCG, PWX11A*, and *IGF1R* on *GAC A4* are seen in *GAC A5*. This result is consistent with the Nakatani model proposing that the three linkage groups shown in Figure 2 were generated through the 2R.

### Subtype-specific motifs in vertebrate GnRHRs

**Figure 3:**
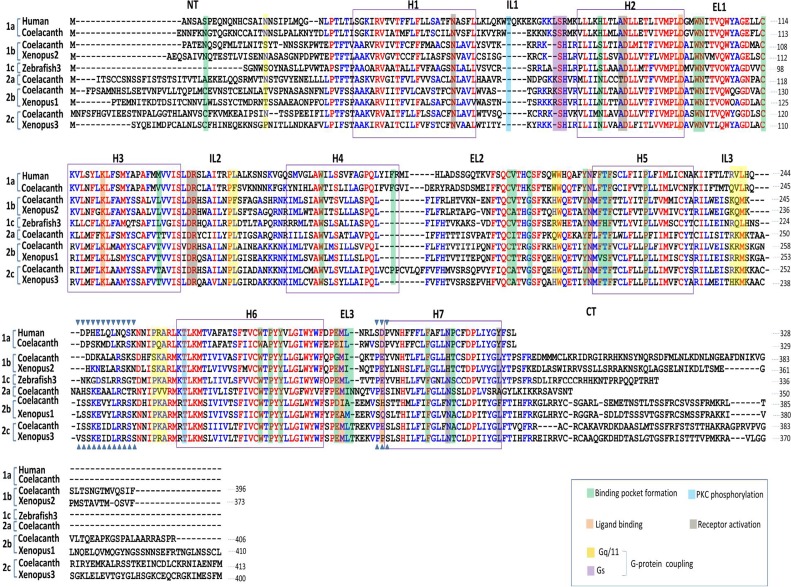
Sequence alignment of coelacanth GnRHRs with vertebrate GnRHRs that represent each subtype. The five coelacanth GnRHRs were aligned with human GnRHR1a, *Xenopus* GnRHR1b, zebrafish GnRHR1c, *Xenopus* GnRHR2b, and *Xenopus* GnRHR2c. Residues with 50% and 90% similarity are indicated in blue and red, respectively. Predicted N terminal (NT), transmembrane helix (H), intracellular loop (IL), extracellular loop (EL) and C-terminal (CT) domains are indicated. The putative residues involved in ligand binding, binding pocket formation, receptor activity, PKC phosphorylation, and G protein coupling are illustrated in different colors. ▾ indicates the subtype-specific motifs.

In a search for new GnRHR genes we identified five new genes in coelacanth. Our synteny and phylogenetic analysis revealed that one of these coelacanth GnRHR genes corresponds to mammalian type I GnRHR (the 1a subtype in this study) and three genes correspond to non-mammalian GnRHRs GnRHRn1 (2b), GnRHRn2 (1b), and GnRHRn3 (2c) according to our previous classification [13]. Surprisingly, one coelacanth GnRHR (2a) is unique and cannot readily be compared with other vertebrate GnRHRs. The five coelacanth GnRHRs were aligned with human 1a, *Xenopus* 1b, 2a, 2b, 2c, and zebrafish 1c GnRHRs (Figure 3). Alignment for all amino acid sequences of GnRHR examined in this study is shown in Figure S1. Sequence alignment analysis revealed that all subtypes of GnRHRs share many critical residues involved in ligand binding, binding pocket formation, G protein coupling, and receptor activation (Figure 3). The major difference between types 1 and 2 is found in intracellular loop 3 (IL3). The type 2 receptors have a longer IL3 that contains a highly conserved SKExxLRr/cS motif. The type 1 receptors have subtype-specific residues in IL3: the 1a subtype has a Tr/qVLh/rQDP motif, the 1b subtype contains KQm/lK motif, and the 1c subtype possesses a slightly longer IL3 without pronounced sequence conservation (Figure S1). Because IL3 is involved in G-protein coupling, receptor desensitization, and internalization [31], [32], this subtype specificity may result in differences in signaling cascades and down-regulation among receptors. Extracellular loop 3 (EL3) also contains subtype-specific motifs: Sd/eP for 1a, PEY for 1b and 1c, Pe/pS for 2a and 1c, and Sh/qS for 2b. These motifs are known to be involved in ligand specificity [33]-[36]. Major differences between subtype 1a mammalian GnRHR and other subtype receptors are the absence/presence of the cytoplasmic tail and residue Asn^2.50^/Asp^2.50^ in transmembrane helix 2 (TMH2) [37]-[40]. Coelacanth GnRHR1a possesses specific characteristics present in mammalian type I GnRHR (the 1a subtype) – it lacks the cytoplasmic tail and contains Asn^2.50^ in TMH2. Thus, coelacanth GnRHR 1a may be an ortholog of mammalian type I GnRHR.

### Ligand selectivity of coelacanth GnRHR 1a

**Figure 4:**
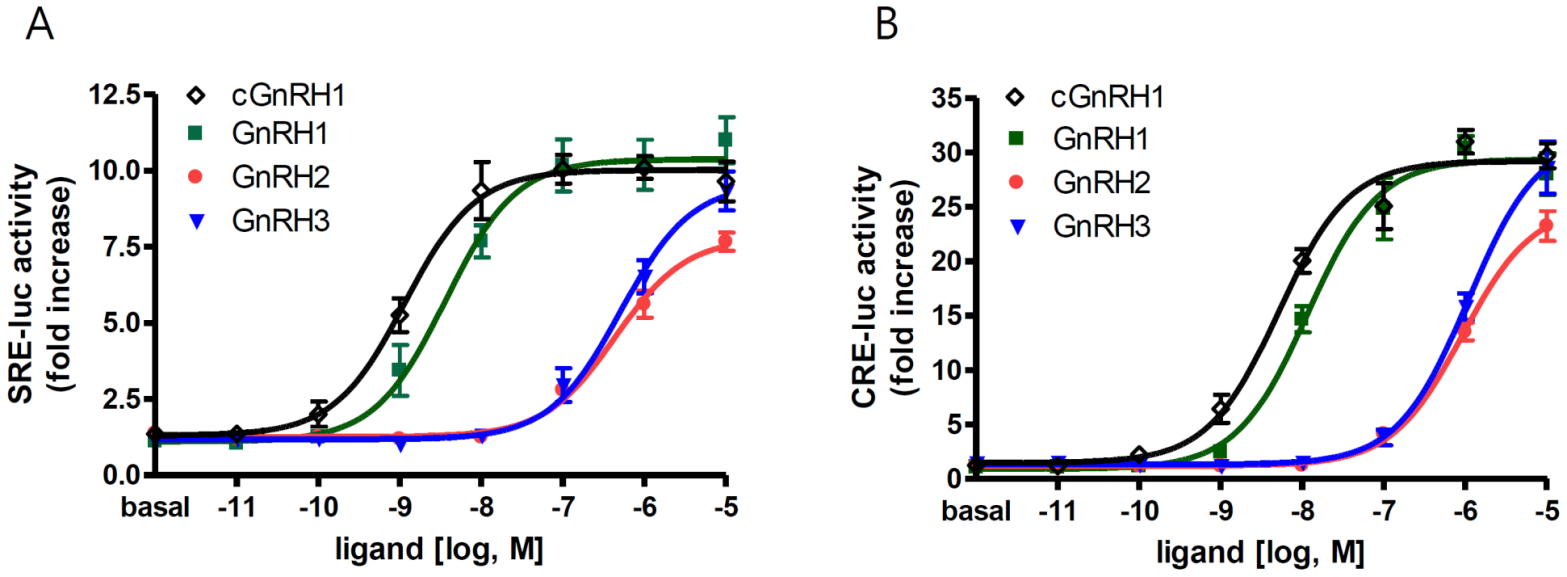
Activity of GnRH peptides with coelacanth GnRHR 1a. Plasmid containing coelacanth GnRHR 1a sequence was co-transfected into HEK293T cells with SRE-luc (***A***) or CRE-luc (***B***) vector in 48-well plates. Forty-eight hours after transfection, cells were treated with the indicated concentrations of peptides (◊ coelacanth (c) GnRH1; ▪ GnRH1; • GnRH2; ▾ GnRH3) for 6 h, and luciferase activity was examined.

To further examine functional orthology between coelacanth GnRHR 1a and mammalian type I GnRHR, we examined the ligand selectivity of coelacanth GnRHR 1a. HEK293 cells were transfected with plasmid containing coelacanth GnRHR 1a and the reporter vector SRE-luc or CRE-luc. Cells were then treated with human GnRH1, coelacanth GnRH1, chicken GnRH2, or salmon GnRH3. In response ligand stimulation, cells transfected with coelacanth GnRHR 1a showed an increase in both SRE-luc and CRE-luc activities, indicating that coelacanth GnRHR 1a may be coupled to both G_q/11_- and G_s_-mediated signaling pathways (Figure 4). Coelacanth GnRHR 1a responded best to human and coelacanth GnRH1 but poorly to GnRH2 and GnRH3, indicating that coelacanth GnRHR 1a has highest affinity for GnRH1, as does the mammalian type I receptor [33], [34].

## Discussion

The evolutionary relationship and history of a gene family can be deciphered by conventional phylogenetic analysis and synteny analysis of chromosomal regions containing members of the gene family [5], [8],[13]. The accumulation of genome sequence information for various invertebrate and vertebrate species has facilitated entire genome comparisons of evolutionarily distant taxa, allowing reconstruction of hypothetical chromosomes of vertebrate or chordate ancestors [6], [7]. Recently, relocating gene family members on these reconstructed vertebrate ancestral linkage groups was found to be a complementary tool to examine the evolutionary history of a gene family [14], [16]. This method is particularly useful for a gene family with a large number of paralogs that arose before WGD [14]. Because conventional small-scale synteny covers relatively short chromosomal regions, neighbor genes located far from the gene of interest on the chromosome or genes on other chromosomes are often excluded from the analyses. In contrast, the size of the reconstructed ancestral linkage group is much greater than that used for small-scale synteny and the ancestral linkage group also includes genes clusters that translocated to other chromosomes [14], [16]. However, this method suffers from poor resolution in some ancestral linkage groups due to either inaccurate reconstruction because of massive chromosome rearrangements, or a single gene translocation that caused it to move from its authentic chromosomal fragments during or after 2R [14], [16]. Thus, combining phylogenic and syntenic analyses is necessary for a better understanding of the evolutionary relationship and history of a gene family [14], [16].

By applying the above approaches, we were able to propose an evolutionary history of the GnRHR family in vertebrates. In our previous study, we classified vertebrate GnRHRs into four subtypes, one mammalian 1 subtype (m1) and three non-mammalian subtypes (n1, n2, and n3) [13]. In the present study, through identification of a novel subtype of GnRHR and improved synteny methods, we propose the presence of six subtypes (1a–2c) of GnRHRs in vertebrates. The m1 subtype is equivalent to the 1a subtype that includes a coelacanth GnRHR. The n1 and n2 subtypes are largely shuffled in the present study (Table 1). The tetrapod n2 matches with 1b, whereas teleost n2 belongs to 2b. The tetrapod n1 and teleost n1 are included in the 2b subtype. The n3 group splits into 1c (teleost receptors) and 2c (tetrapod receptors). This confusion is largely due to multiple local duplications of GnRHR before 2R (and after 3R in teleosts) followed by either lobed-finned fish- or ray-finned fish-specific loss of the subtype gene. For example, the n1 and n2 subtypes (1b and 2b in this study) emerged via a local duplication on the same chromosome before 2R, and additional subtypes 2b′ and 2b″ in teleosts arose through 3R and a subsequent local duplication. Thus, our previous synteny failed to explore the fine mechanisms of the emergence of GnRHR subtypes through genome duplications and local duplications [13]. A new GnRHR found in coelacanth is likely to belong to a new subtype 2a.

The phylogenetic trees constructed in our study and by others reveal that the 1a subtype receptors are phylogenetically closer to invertebrate receptors than to other receptors [27] suggesting that, among vertebrate receptors, the 1a subtype receptors are probably closer to an ancestral form of GnRHR. Interestingly, amphioxus has four GnRHRs, two of which are similar to the protostomian GnRHR found in *Drosophila* and *C. elegans* whereas the other two are similar to vertebrate GnRHR [27], [41]. Indeed, a recent study showed that the vertebrate-like amphioxus GnRHRs exhibited a high affinity to vertebrate GnRHs but not to protostomian GnRHs, whereas protostomian-like amphioxus GnRHRs had poor affinity to vertebrate GnRHs [27]. Thus, amphioxus contains two GnRHR lineages, vertebrate-like GnRHR and protostomian-like GnRHR. The protostomian GnRHRs were not found in the basal vertebrate lamprey. Instead, lamprey contains three receptors which are likely to belong to type 2 receptors. As the scaffolds containing lamprey GnRHRs are short and do not have any neighbors, syntenic analysis to explore the evolutionary position of these genes is impossible.

Our chromosome analysis showed that three pairs of the subtypes (1a–2a, 1b–2b, and 1c–2c) are present on three paralogous chromosomal regions. Although pairs of the subtype are on the same chromosomes, members in each pair are phylogenetically distant; instead, the phylogeny tree shows a close relationship among type 1 subtypes (1a, 1b, and 1c) and type 2 subtypes (2a, 2b, and 2c). This indicates that a local duplication of GnRHR occurred before 2R and that consecutive chromosome duplications have contributed to the generation of three pairs. The fourth pair was probably lost after 2R. There is accumulating evidence for local duplication gene families before 2R. In the case of somatostatin and its paralogous genes cortistatin, urotensin II, and urotensin II-like peptide, two pairs of the genes are on two paralogous chromosomes [11]. The neuropeptide Y receptor (NPYR) triplet is proposed to have existed before 2R [9]. In addition, prolactin-releasing hormone receptors, which are phylogenetically very close paralogous receptors for NPYR, are also in the near vicinity of NPYR on the same chromosomes [10]. The secretin-like receptors and their peptides genes are also clustered on paralogous chromosomes [14], showing that paralogous genes that arose through local duplications reside in the near vicinity on the same chromosomes or on reconstructed ancestral chromosomes.

The origin of type 1 mammalian GnRHR has been questioned because orthologs of the mammalian receptor have not been found in other vertebrates and the structure of the mammalian receptor is quite different from that of other receptor subtypes [13], [27], [42]. In our study, we found that a coelacanth receptor is similar to the mammalian receptor based on amino acid sequence. Like mammalian GnRHR, this coelacanth receptor lacks the C-terminal cytoplasmic tail, has conserved Asn^2.50^/Asp^7.49^ residues in TMH2 and THM7, and harbors an Sd/eP motif in ECL3. The presence or absence of the C-terminal cytoplasmic tail is important for receptor down-regulation and internalization [37]. For example, addition of the C-terminal cytoplasmic tail to mammalian GnRHR greatly enhances the internalization and desensitization rates [37]. Furthermore, the C-terminal cytoplasmic tail is likely to be involved in signaling pathway. Deletion of the C-terminal tail from bullfrog GnRHR-1 remarkably decreased its ability to induce G_s_-mediated adenylyl cyclase (AC)/protein-kinase A (PKA) signaling. In contrast, addition of this motif to mammalian GnRHR improved its ability to induce the AC/PKA-linked signaling pathway [38]. Interaction of the Asn^2.50^ residue in TMH2 with the Asp^7.49^ residue in TMH7 of mammalian GnRHR is essential for receptor conformation and activation whereas non-mammalian receptors have Asp^2.50^/Asp^7.49^ in both TMH2 and TMH7 [39], [40]. The GnRH binding residues (i.e. Asp^98^, Asn^102^, Lys^121^, Asn^212^, Trp^280^, Trp^289^, and Tyr^290^) of mammalian GnRHR-1 are highly conserved among vertebrate GnRHRs [43]–[47]. Thus, a mammalian GnRHR-specific motif, the Sd/eP motif in ECL3, was considered to be important for high affinity toward GnRH1 [33]–[35]. Indeed, our study shows that the coelacanth receptor with the SDP motif responded best to human and coelacanth GnRH1 but poorly to GnRH2 and GnRH3. In particular, differences in the specific domains (SD/EP, PEY, and PPS) at the ECL3 have been used for classification of GnRHR [36], [42]. Taken together, these findings indicate that the coelacanth receptor is a functional ortholog of the mammalian type 1 GnRHR, thus the 1a subtype receptor is no longer unique to mammals.

In summary, the evolutionary history of the GnRHR represents repetitions of continuous duplications and losses within a gene family during metazoan evolution. In particular, during emergence of early vertebrates, at least two GnRHRs appear to have been generated through local duplication. This doublet was amplified as three pairs through WGD, followed by loss of redundant members before the divergence of ray-finned and lobed-finned fish. Evolution of GnRHR in teleosts is more complicated than that of tetrapods due to teleost-specific 3R, local duplication after 3R, and loss of genes before or after 3R. Because of the complexity of GnRHR evolutionary history, a combination of phylogenetic analysis, small-scale synteny, and relocating gene family on the reconstructed ancestor chromosome is required for better understanding of the evolutionary history of the gene family. This issue will be further clarified when improved genome data of other vertebrate and invertebrate species are available.

## Materials and Methods

### Phylogeny of the GnRHR family

ENSEMBE (http://www.ensembel.org) and GenBank (http://www.ncbi.nlm.nih.gov/Entrez/) databases were used to retrieve all annotated amino acid sequences for GnRHR of vertebrates including lamprey, spotted gar, zebrafish, medaka, fugu, stickleback, tetraodon, coelacanth, anole lizard, *Xenopus*, chicken, mouse, and human, and of invertebrates including *C. elegans*, *Drosophila*, *Ciona*, and amphioxus [26], [27], [30], [41], [48]–[51]. Orthologous or paralogous genes from vertebrate and invertebrate species were identified through research tools provided by the ENSEMBEL database. To obtain additional sequences other than the well-annotated query sequences, a TBLASTN algorithm was performed against a genome database of selected species [14]. The phylogenetic analysis of GnRHR sequences was accomplished as previously described [14]. The MUSCLE algorithm with default settings was used to align the sequences as implemented in MEGA 5.05. A maximum likelihood phylogenetic tree was created following the Jones-Taylor-Thornton model with a confidence level of 100 replicate bootstrap samples.

### Synteny analysis and location of GnRHR genes on reconstructed ancestral linkage group

For synteny analysis, contig views of genome regions surrounding the GnRHR loci were compared. The exact map location of orthologs/paralogs of neighboring genes was obtained from the ENSEMBLE database [14]. Next, the chromosomal segments of three taxa (medaka, chicken, and human) with a reliable synteny were matched with vertebrate ancestral linkage groups according to the Nakatani model [6] as described previously [14], [16]. The Nakatani model provides vertebrate ancestral linkage blocks displayed on individual human chromosomes compared with the corresponding chromosome locations of medaka, chicken, and mouse [6]. Comparison of results from each taxon resolved the locations of the gene blocks at consecutives stages of vertebrate genome evolution.

### Chemicals and hormones

All chemicals were obtained from Sigma-Aldrich (St. Louis, MO, USA) unless otherwise stated. Restriction enzymes were obtained from New England Biolabs (Ipswich, MA, USA). Human (hu) GnRH1, coelacanth GnRH1 (pyroEYWSYDLRPG-NH2), chicken GnRH2, and salmon GnRH3 were synthesized by AnyGen (Gwangju, Korea). The purities of the synthesized peptides were greater than 98% based on high-performance liquid chromatography. All peptides were dissolved in distilled water and then diluted in media to the desired working concentrations.

### Plasmid constructs

The pcDNA3.1 expression vector was purchased from Invitrogen Corp. (San Diego, CA, USA). The CRE-luc vector containing four copies of the cyclic AMP-responsive element (CRE; TGACGTCA), and the SRE-luc vector containing a single copy of the serum response element (SRE, CCATATTAGG) were purchased from Stratagene (La Jolla, CA, USA). The cDNA for coelacanth GnRHR 1a was synthesized by GenScript (Piscataway, NJ, USA). The cDNA was incorporated into the *EcoR*I and *Xho*I sites of pcDNA3.1 and the resulting construct was verified by sequencing.

### Cell transfection and luciferase assay

HEK293T cells (American Type Culture Collection, Manassas, VA, USA) were maintained in Dulbecco's modified Eagle's medium (DMEM) supplemented with 10% fetal bovine serum, 100 U/ml penicillin G, and 100 µg/ml streptomycin (Invitrogen). Cells were seeded in 48-well plates at a density of 1.4×10^4^ cells per well 1 day before transfection. A mixture containing 75 ng of CRE-luc (or SRE-luc), 75 ng of the coelacanth GnRHR 1a plasmid, and 2 µl of Effectene reagent (Qiagen, Chatsworth, CA, USA) was prepared and added to each well according to the manufacturer's instructions. Approximately 48 h after transfection, cells were treated with the respective ligands for 6 h. For SRE-luc analysis, cells were maintained in serum-free DMEM for at least 16 h before ligand treatment. Cells were lysed by addition of 100 µl lysis buffer and the luciferase activity in 40 µl of cell extract was determined on a luciferase assay system according to the standard protocol for the Synergy 2 Multi-Mode Microplate Reader (BioTek, Winooski, VT, USA).

### Data analysis

Data analysis was performed by nonlinear regression with a sigmoidal dose-response. The agonist concentrations that induced half-maximal stimulation (EC_50_) were calculated with GraphPad PRISM4 software (GraphPad Software Inc., San Diego, CA, USA). All data were presented as mean ± S.E. of at least three independent experiments.

## Supporting Information

Figure S1 Sequence alignment of vertebrate GnRHRs. GnRHRs from human, coelacanth, anole, chicken, *Xenopus*, zebrafish, medaka, tetraodon, fugu, and stickleback are shown. Residues with 50% and 90% similarity are indicated in blue and red respectively. Predicted N terminal (NT), transmembrane helix (H), intracellular loop (IL), extracellular loop (EL), and C terminal (CT) domains are indicated.
(PDF)

Table S1 The accession numbers for NCBI and ENSEMBL of *C. elegans*, *Drosophila*, *Ciona*, and amphioxus GnRHRs are given beneath receptor's name as well as gene location in brackets.
(PDF)
