# Supplementary material for: Local Duplication of Gonadotropin-Releasing Hormone (GnRH) Receptor before Two Rounds of Whole Genome Duplication and Origin of the Mammalian GnRH Receptor
Source: PLoS One. 2014 Feb 3;9(2):e87901. doi: 10.1371/journal.pone.0087901 (PMC3912137; doi:10.1371/journal.pone.0087901)
Supplement: Figure S1 — Sequence alignment of vertebrate GnRHRs. GnRHRs from human, coelacanth, anole, chicken, Xenopus, zebrafish, medaka, tetraodon, fugu, and stickleback are shown. Residues with 50% and 90% similarity are indicated in blue and red respectively. Predicted N terminal (NT), transmembrane helix (H), intracellular loop (IL), extracellular loop (EL), and C terminal (CT) domains are indicated. (PDF) [file pone.0087901.s001.pdf]

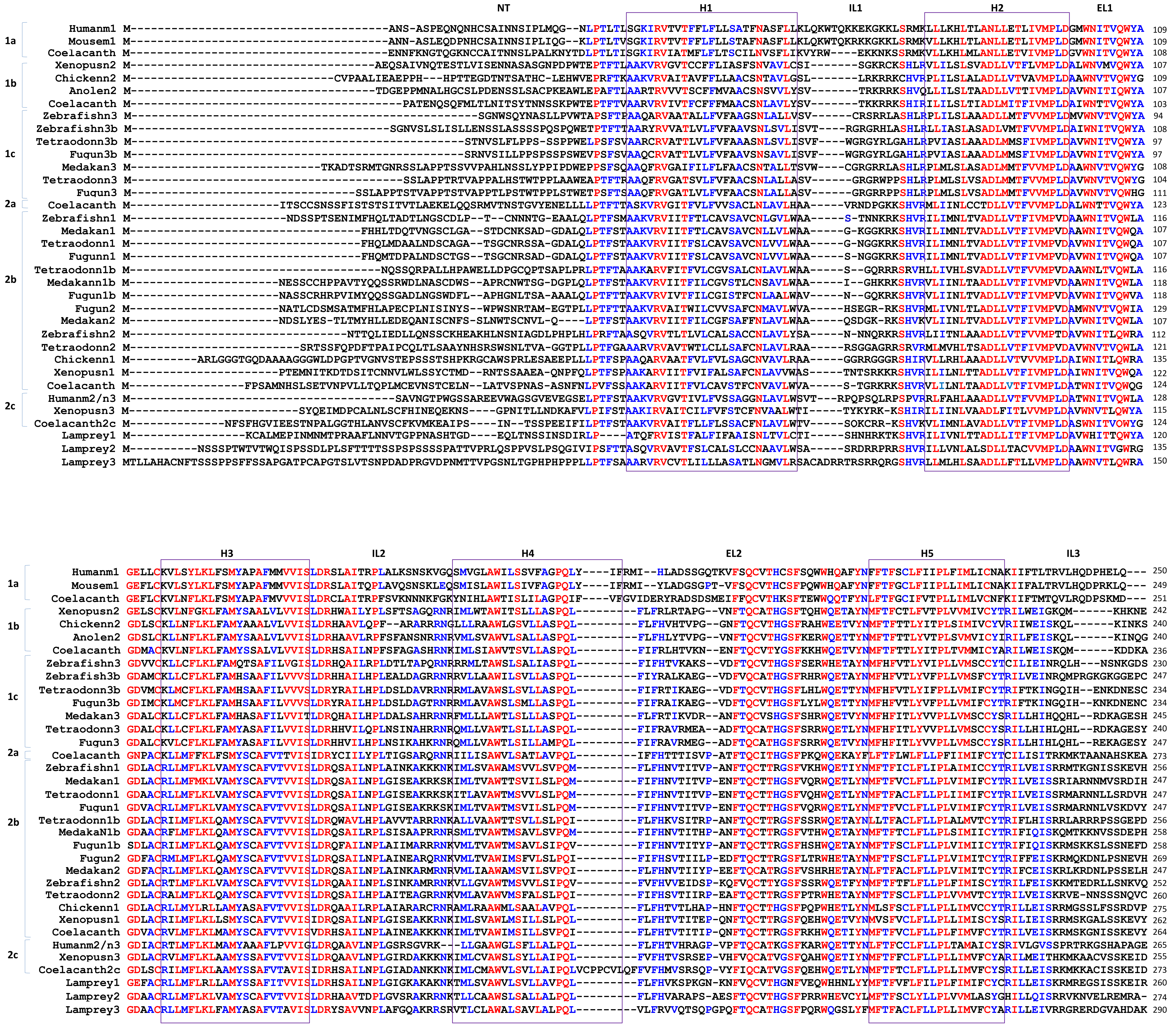

NT
H1
IL1
H2
EL1
1a
1b
1c
2a
2b
2c
Humanm1
Mousem1
Coelacanth
Xenopusn2
Chickenn2
Anolen2
Coelacanth
Zebrafishn3
Zebrafishn3b
Tetraodonn3b
Fugun3b
Medakan3
Tetraodonn3
Fugun3
Coelacanth
Zebrafishn1
Medakan1
Tetraodonn1
Fugunn1
Tetraodonn1b
Medakann1b
Fugun1b
Fugun2
Medakan2
Zebrafishn2
Tetraodonn2
Chickenn1
Xenopusn1
Coelacanth
Humanm2/n3
Xenopusn3
Coelacanth2c
Lamprey1
Lamprey2
Lamprey3
H3
IL2
H4
EL2
H5
IL3

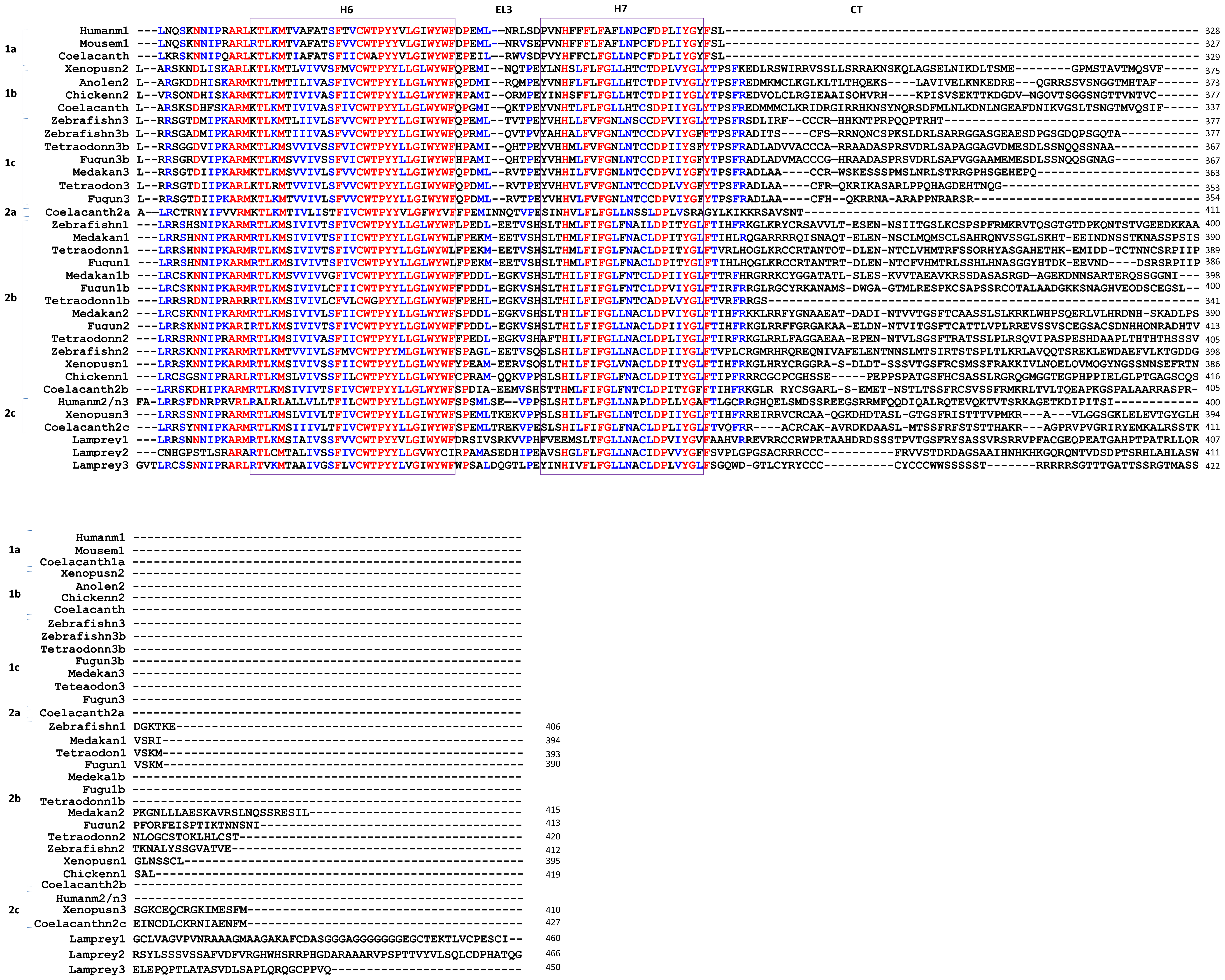
H6
EL3
H7
CT
1a
1b
1c
2a
2b
2c
Humanm1
Mousem1
Coelacanth
Xenopusn2
Anolen2
Chickenn2
Zebrafishn3
Zebrafishn3b
Tetraodonn3b
Fugun3b
Medakan3
Tetraodon3
Fugun3
Coelacanth2a
Zebrafishn1
Medakan1
Tetraodonn1
Fugun1
Medakan1b
Fugun1b
Tetraodonn1b
Medakan2
Fugun2
Tetraodonn2
Zebrafishn2
Xenopusn1
Chickenn1
Coelacanth2b
Humanm2/n3
Xenopusn3
Coelacanth2c
Lamprey1
Lamprey2
Lamprey3
Coelacanth1a
Medekan3
Teteaodon3
Tetraodon1
Medeka1b
Fugu1b
Coelacanthn2c
