# Supplementary material for: Local Duplication of Gonadotropin-Releasing Hormone (GnRH) Receptor before Two Rounds of Whole Genome Duplication and Origin of the Mammalian GnRH Receptor
Source: PLoS One. 2014 Feb 3;9(2):e87901. doi: 10.1371/journal.pone.0087901 (PMC3912137; doi:10.1371/journal.pone.0087901)
Supplement: Table S1 — The accession numbers for NCBI and ENSEMBL of C. elegans, Drosophila, Ciona, and amphioxus GnRHRs are given beneath receptor's name as well as gene location in brackets. (PDF) [file pone.0087901.s002.pdf]

Table S1. The accession numbers for NCBI and ENSEMBL of *C. elegans*, *Drosophila*, *Ciona*, and amphioxus GnRHRs are given beneath receptor's name as well as gene location in brackets.

	Invertebrate Species			
	**C.elegans**	**Drosophila**	***Ciona intestinalis***	***Amphioxus***
**Receptors**	**GnRHR**<br>NP_001249720<br>(Ch1)	**GnRHR1**<br>FBgn0025595<br>(Ch2L)	**GnRHR1**<br>NP_001027765<br>(Ch3)	**GnRHR1**<br>EU433377<br>(Scaffold 15)
		**GnRHR2**<br>FBgn0036278<br>(Ch3L)	**GnRHR2**<br>NP_001027656<br>(Ch3)	**GnRHR2**<br>EU433378<br>(Scaffold 15)
			**GnRHR3**<br>NP_001165352<br>(Ch3)	**GnRHR3**<br>EU433379<br>(Scaffold 629)
			**GnRHR4**<br>NP_001165351<br>(ScaffoldHT000157)	**GnRHR4**<br>FJ426561<br>(Scaffold 15)
